# Traditional knowledge of medicinal plants on Gau Island, Fiji: differences between sixteen villages with unique characteristics of cultural value

**DOI:** 10.1186/s13002-021-00481-w

**Published:** 2021-10-11

**Authors:** Kana Miyamoto, Hiroshi Ehara, Randolph Thaman, Joeli Veitayaki, Takehito Yoshida, Hikaru Kobayashi

**Affiliations:** 1 Department of General Systems Studies, Graduate School of Arts and Sciences, The University of Tokyo, Bldg. 15 room 509, 3-8-1, Komaba, Meguro-Ku, Tokyo 153-8902 Japan; 2 School of Geography, Earth Science and Environment, Faculty of Science, Technology and Environment, The University of the South Pacific, Private Bag, Laucala Campus, Suva, Fiji; 3 International Center for Research and Education in Agriculture, Nagoya University, Furo-cho, Chikusa-ku, Nagoya, 464-8601 Japan; 4 School of Marine Studies, Faculty of Science, Technology and Environment, The University of the South Pacific, Laucala Bay Road, Suva, Fiji; 5 Research Institute for Humanity and Nature, 457-4 Motoyama, Kamigamo, Kita-ku, Kyoto, 603-8047 Japan

**Keywords:** Fiji, Gau Island, Social and ecological factors, Sustainable practice of medicinal herbal plants, Traditional resource management (TRM)

## Abstract

**Background:**

Traditional resource management (TRM) systems develop depending on local conditions, such as climate, culture, and environment. Most studies have focused on the TRM system itself, excluding the people who manage the system, and the relationship between the system and the people. The use of resources and people is intimately linked through the practice of TRM systems on Gau Island and this relationship needs to be understood to advance sustainable resource use.

**Methods:**

A survey was conducted on the use of medicinal plants on Gau Island, Fiji. Interviews were conducted from September 2013 to January 2015 with knowledgeable members of each community. The types of plants, prescriptions, and health problems were documented, and social and ecological factors affecting the sustainability of TRM of medicinal plants used in each of the 16 villages were statistically analysed by linear regression analysis.

**Results:**

A total of 58 medicinal plants used on a daily basis to treat 27 health problems were identified on Gau. Two medicinal plants, Botebotekoro (*Ageratum conyzoides*) and Totodro (*Centella asiatica*), were used in all districts to treat various health problems. There were contrasts between the villages in the medical lore and prescriptions, and villages often used different traditional treatments than others for the same ailment; therefore, the status and knowledge of medicinal plants have developed distinctly in each village. Geographical and social factors have been suggested as possible reasons for the differences in regional resource utilisation among villages. Statistical analysis of the relationship between the state of TRM and social and ecological factors suggest that community solidarity has a positive impact on the sustainable practice of TRM. This study showed that traditional practices simultaneously contribute to the conservation of the natural environment and the binding of communities.

**Conclusions:**

The results highlight the importance of understanding how TRM systems can contribute to the conservation of the natural environment. Cultural activities are essential to raise community solidarity, which has led to the sustainable use of natural resources. This suggests that merely documenting the use of medicinal plants is not enough to ensure that the skills and knowledge are passed down to the next generation.

**Supplementary Information:**

The online version contains supplementary material available at 10.1186/s13002-021-00481-w.

## Background

Herbal medicines have been an important resource for the maintenance of human health throughout history [1]. Until recently medicinal plants have played crucial roles on Gau Island, Fiji, as there is limited access to external medical support on the island. Although the Fijian healthcare system is free [2] and Gau now has a local doctor and a few nursing stations, there is still a demand for medicinal plants. Village elders collect medicinal plants for the treatment of less serious diseases and general health issues; however, modernisation attracts younger generations and influences their view of medical services. There is a trend towards the use of modern medicinal drugs as opposed to traditional herbal medicines, and younger generations are losing knowledge about the use of medicinal plants. In addition, there are signs that the land is being unsustainably used, with forests that are important sources of plants used in traditional medicine being cleared to make way for agricultural cash crops. Deforestation causes increased biodiversity loss, which has jeopardised traditional healthcare systems, and shifted the dependence of people from ethnomedicine to modern medicine [3].

Traditional Resource Management (TRM) is a sustainable practice for managing natural resources to secure livelihood ([4], as cited [5]) and is common worldwide. A study on culture and marine conservation in Fiji described that TRM practices form an integral part of the culture, knowledge, and tradition of the local people [6]. The same situation may apply to TRM of medicinal plants in Gau, as TRM is also recognised as an ancient practice for Gau islanders.

TRM also plays a significant role in improving the condition of life on the island and has allowed people to continue to live in the communities throughout history [7]. Gau’s TRM system provides people with valuable lessons on sustainable resource use and survival within their natural surroundings [8]. For example, TRM provides not only the skill and knowledge for sustainable use of natural resources, but also opportunities for participating in cultural events and community activities, and the teaching of traditional roles, which are important factors in forming personal ethical principles, attitudes, and cultural beliefs, which in turn influence the level of sustainability.

TRM on Gau is led by key persons such as the chief, Turaga ni Koro (village leader), and ex-Turaga ni Koro, although any villager can participate in decision-making in the TRM system. They can express their opinions and provide information which may be important for the protection of medicinal plants. In addition, people value the opinions of medicine women and men, and they are still revered on the island. Hence, the TRM system of medicinal plants is still efficiently practiced.

Most ethnobotanical studies on traditional medicinal plants have focused on their role in primary health care or their significance to traditional knowledge and skills. Although this continues to be a major research interest [9], it is also necessary to determine and understand the contribution of TRM systems to sustainable communities, because TRM is involved in various ways, not only for managing natural resources themselves but also for organising social factors such as resource distribution (market system) and the contribution of users (i.e. participation and responsibilities) to sustainability. Jentoft et al. [10] highlighted the study of cultural roles as important because resource management contributes to the organisation of the community’s activities, by enabling people to live in a rich natural environment, and as Veitayaki et al. [6] demonstrated, manage their environmental resources and pursue sustainable development activities. Hence, gaining a better understanding of the mechanisms underlying such systems, including the various parts that TRM plays in a community, can facilitate the survival of the entire system for future generations; therefore, this key subject cannot be ignored. As Bisht and Sharma [11] describe, TRM has evolved through co-existence, interactions, and a reciprocal response between society and nature, since conservation of the natural system while ignoring the social system has created severe imbalances in the overall ecosystem of the area (p. 97). Kunwar et al. [12] also described that the lack of an organised sustainable cultivation based on scientific data and an inadequate awareness of social factors influencing the plant use and market can cause a decline in the proper management of traditional medicine and consequently a reduction in the plants and indigenous knowledge on resource use.

From our observations, each of the villages on Gau uses different combinations and preparations of medicinal plants. Such sustainable use of resources is considered to vary among villages and depends on social and ecological factors such as beliefs, availability of land, availability of medicinal plant species, population, and the state of the local economy. The use of resources is intimately linked to the population through locally developed practices of traditional resource management (TRM); therefore, an overarching objective of this study was to improve the sustainability of natural resources used on Gau Island by identifying key social and ecological factors that influence the TRM system, based on the concept proposed by McGinnis and Ostrom [13]. This article presents the results of a survey conducted to document traditional medicinal knowledge and skills, as well as to gain an understanding of the TRM system on Gau.

## Methods

### Study sites

**Fig. 1:**
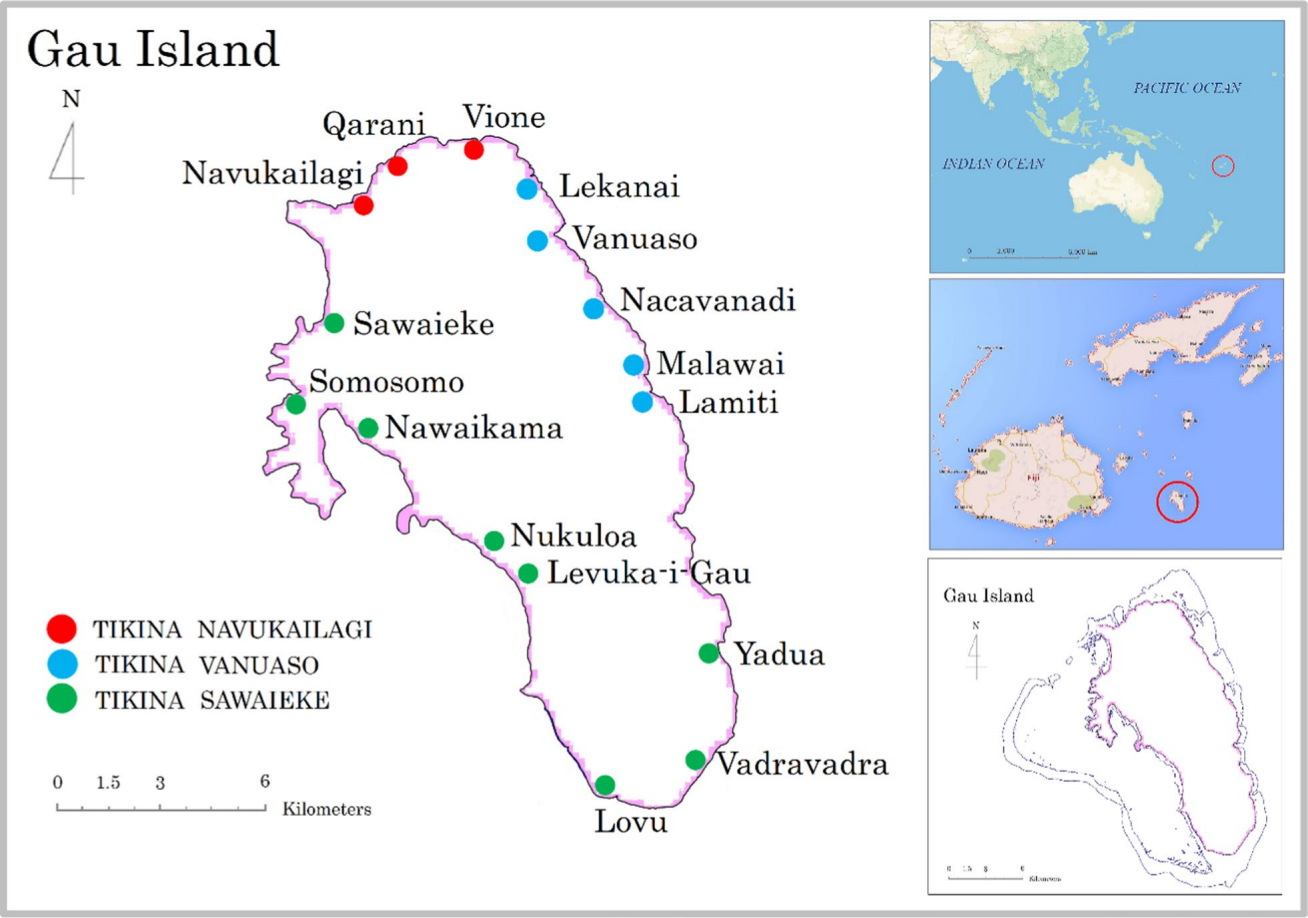
Map of Gau Island. Gau Island is the fifth largest island in Fiji, located approximately 85 km east of the Suva Peninsula

Gau Island is the fifth largest island of the Fiji group and is located in the Lomaiviti archipelago, approximately 85 km east of the Suva Peninsula (Fig. 1). Gau is recognised for its beauty and nature, and is the home of the endemic Fiji petrel (*Kacau Ni Gau; Pseudobulweria macgillivrayi*), which is commonly known to nest in Gau, and considered to be a critically endangered species according to the IUCN Red List of Threatened Species [14]. The isolation of Gau from the main islands has hindered development but has preserved its rich natural environment. Gau islanders maintain a semi-subsistent lifestyle and have no main electricity supply. A virgin cloud forest in the middle of the island covers approximately two-thirds of the island (300 km^2^), which is shared by 16 villages, each of which manages the part of the forest assigned to that village. Some villages have cleared the forest to expand the area of agricultural land for the production of cash crops to increase income and improve their quality of life, while others have focused on conserving the rich natural environment. Rather than clearing the forest, some villages have expanded the amount of agricultural land available by planting pine and leguminous trees to improve the fertility of unproductive barren land.

Gau is divided into three *Tikina* (districts) (see Table 1): (1) *Tikina Vanuaso* in the northeastern region of the island (including the villages Lekanai, Vanuaso, Nacavanadi, Malawai and Lamiti); (2) *Tikina Navulailagi* (Navukailagi, Qarani, and Vione); and (3) *Tikina Sawaieke* (Yadua, Vadravadra, Lovu, Levuka-i-Gau, Nukuloa, Nawaikama, Somosomo and Sawaieke), the latter containing two satellite communities [7]. The island has a population of approximately 2200 people, spread among the 16 villages.

**Table 1:** Name of villages in Gau Island with population, number of plants used in each village and number of wounds/symptoms treated by medicinal plants

No	Tikina	Village	Population	Number of plant used in each village	Number of wounds/symptoms
1.	Tikina Navukailagi	Navukailagi	94	6	3
2.		Qarani	133	10	4
3.		Vione	99	5	3
4.	Tikina Vanuaso	Lekanai	98	10	5
5.		Vanuaso	150	4	4
6.		Nacavanadi	170	6	2
7.		Malawai	154	3	3
8.		Lamiti	224	7	4
9.	Tikina Sawaieke	Yadua	74	6	3
10.		Vadravadra	140	8	3
11.		Lovu	135	9	4
12.		Levuka-i-Gau	128	5	2
13.		Nukuloa	116	3	3
14.		Nawaikama	292	3	2
15.		Somosomo	117	3	3
16.		Sawaieke	164	3	1

Qarani, which is in the central region of Gau, has a medical centre with a doctor, and Nacavanadi has a nursing station containing only nurses. Gau has only one high school at Nawaikama, the village with the largest population with approximately 292 people; Yadua, Navukailagi, Vione and Lekanai have the smallest populations of the 16 villages, with close to 100 people each.

### Interview and data collection

The survey was conducted in 16 villages from September 2013 to January 2015 to collect information on medicinal plants, their locations and prescribed uses, as a part of the JICA Grass-roots Cooperation Programme (2013–2015) for the sustainable development and governance of Gau Island in Fiji. Most of the reported knowledge focused on common plants used for general health remedies, as some of the special medicinal plants have been kept secret [8] by the owners. According to oral histories in the South Pacific, medicinal plants have been used as a spiritual tool, and the related knowledge was kept by chosen family members to consolidate their power to lead the community.

Semi-structured interviews were employed to collect data, and two or three key interviewees from each village participated in the study. In each village, the interviewee was selected based on the recommendation of the chief or village leader. The selection was generally based on those who were well known for providing medicinal plants to the villagers. In some cases, a few middle-aged villagers joined the interviews, whereas most of the informants were older women among the family members. Only one older man from Lovu, who was the chief of the village, participated in the survey. Even though many medicine women are active in Fiji, this situation differs from other regions in Fiji and overseas regions such as Bali, Indonesia. Sujarwo et al. [15] reported that informants are mainly constituted by men. This ratio between men and women as interviewees may be derived from the history of Gau Island. According to villagers, those fleeing battles settled on Gau Island, and there was no one with spiritual power, such as a shaman to provide traditional medicine. Instead, medicine providers were revered within their communities [8], and it is assumed that peace meant closing gaps between women and men.

Each interviewee was asked for their personal information, such as their name and age, and how often they were asked to provide traditional medicines to their communities. In addition, each interview was started after an agreement with each interviewee has been made to share with others information provided during an interview, as this was the first time that this information was shared with other villages on Gau Island.

Medicinal knowledge manifests in different ways, including through an understanding of medicinal plants and preventive and curative health care, as well as cultural definitions of health and illness. The interviewee was asked for the name of the disorder, the plant used for treatment, and its prescribed use. Information gathered on plants included the type, the part of the plant used, the location of that plant, and its relative abundance. The prescribed use was explained verbally and sometimes demonstrated. The data were recorded using a questionnaire (Additional file 1: Appendix A).

The type of plant was recorded to assist in identification. There were four categories: (1) ground vegetation (tall tree: taller than 3 m); (2) ground vegetation (small tree: less than 3 m in height); (3) herbs (herbs, ferns, and grasses); and (4) climbing herbs (vines). Terms such as ‘shrub’ were intentionally avoided to make the interviews easier for villagers to understand. Four plant parts used as medicine were recorded: the stem, leaf, bark, and root.

The interviewees were asked to identify the habitat in which the plant was found. There were five habitat types: (1) mangroves, (2) sea coasts, (3) villages, (4) vegetation (farmland), and (5) virgin cloud forest. Plants, such as vines, growing along the coast, including beaches, were classified as ‘sea coast’, except for mangrove trees. If the plant habitat was inside a village or compound in residential areas, or by a road close to a village, they were classified in the village category. Plants found in and around the vegetation areas of agricultural crops were classified in the vegetation category, and those found in native forest habitats were classified as virgin cloud forest plants.

‘Abundance’ is subjective and it is difficult to collect accurate data on this parameter; therefore, illustrations (Additional file 1: Appendix B) recommended by the University of the South Pacific were used to record abundance data with greater accuracy. The interviewees were asked to assess the abundance level of the vegetation shown in the image. Abundance was recorded on a six-point scale as follows: V = very abundant, A = abundant, C = common, O = occasional, U = uncommon, and R = rare. If a plant was ‘very abundant’, the interviewee chose an illustration full of trees (column on the left side: 80–100% of trees in a space), meaning that they can be found anywhere and anytime in the village. If an interviewee found a plant easily, but felt abundance is less than ‘very abundant’, they chose ‘abundant’ (second column from the left side: 60–80%), and if an interviewee could find a plant with difficulty and abundance was less than ‘abundant’, they chose ‘common’ (third column from the left side: 40–60%). ‘occasional’, ‘uncommon’, and ‘rare’ were chosen when an interviewee took time to locate a plant. If it was considered ‘rare’, an interviewee chose the illustration with only one tree (column in the right side: less than 10% of space is filled with trees), indicating that it is hard to find and sometimes cannot be found anywhere in their village. If an interviewee took time to find a plant but was eventually able to locate one, they chose ‘uncommon’ (second column from the right side: 10–20%), and if an interviewee required time to find a plant but abundance was more than uncommon, they chose ‘occasional’ (third column from the right side: 20–40%).

Plant specimens were not collected as this research aimed for identifying local knowledge of medicinal plants usage on the island, and did not include collection of specimens as we were not permitted to do so by the local administrations. The scientific names of medicinal plants were identified using illustrated reference books, project reports, and websites. The books and reports used were Cambie and Ash [16], Keppel and Gazanfar [17], Whistler [18], Parham [19], and WAI VAKAVITI Fijian Medicine [20]. There is no scientific name for 8 species locally identified. Among them, three are identified as medicinal plants in WAI VAKAVITI Fijian Medicine [20], but no scientific name is given for Daliganirapete, Drau ni molikaro, and Yamenilaione. Other 3 species were recognized and locally named by people on Gau Island but no scientific name can be identified in this study. Rest of 2 species, Drauni lolo is considered the same species with lolo listed in Fijian Medicinal Plants [16], and Kavika vovo is determined as unidentified species [16].

Data for statistical analysis were collected during interviews with the chief or village leaders. Each village records basic information, such as population, births, and crop production. Agricultural land areas were analysed using GPS location data collected during a field survey using a mobile GPS device. The economic value of medicinal plants and solidarity in a community were determined by how each interviewee expressed their feelings on the topics. The perception of whether or not TRM is well-managed in a community was based on a comparison of the frequency between the current situation and the recollection of the situation when the interviewee was younger. If the frequency of providing medicinal plants was considered to be the same as before, this represented a case in which TRM was well managed.

### Statistical analysis

A statistical analysis was conducted to determine the social and ecological factors that affected the state of the TRM system. The relationship between social and ecological factors (independent variables) and the current state of the TRM system (dependent variable) was examined by linear regression analysis using the glm function (logit link and binomial distribution) in the R software [21].

The five independent variables (Additional file 1: Tables S1 and S2) were classified into the four core components, following the social-ecological system framework developed by McGinnis and Ostrom [13]: (1) government system (GS) and (2) actor (A) belonging to the social side, and (3) resource system (RS) and (4) resource units (RU) belonging to the ecological side. The numerical values of agricultural areas, number of units (average), and population were analysed after standardisation in R using the scale() function, and other variables (economic value and community solidarity) took a value of 0 (negative) or 1 (positive) indicating a response of ‘No’ or ‘Yes’, respectively, indicating whether there is economic value of medicinal plants and solidarity in a community.

The dependent variable, the state of the TRM system, was assigned a value of 0 (the TRM system of medicinal plants was not performed well) or 1 (the TRM system of medicinal plants was performed well).

## Results and discussion

### Medicinal plants used on Gau Island

In total, 58 types of medicinal plants belonging to 27 families (Table 2) from 16 villages in Gau were documented. These plant species were used to treat 27 health conditions (Table 3).

**Table 2:** Medicinal plants species recorded in the study

No	Fijian local name	Scientific name (family)	Identification
1.	Baka ni viti	*Ficus obliqua Forst. f.* (Moraceae)	[16]
2.	Batimadramadra	*Bidens pilosa* L. (Asteraceae)	[16]
3.	Botebotekoro	*Ageratum conyzoides* L. (Asteraceae)	[16]
4.	Botebotekoro tagine	*Ageratum conyzoides* L. (Asteraceae)	[18]
5.	Bobo	*Desmodium adscendens* (SW.) DC. [syn. D. *trichocaulon* DC.] (Fabaceae)	[16]
6.	Bovo (Vobo)	*Mussaenda raiateensis* J.W.Moore (Rubiaceae)	[16]
7.	Danidani	*Polyscias fruticosa* (L.) Harms. (Araliaceae)	[16]
8.	Daliganirapete	(unidentified) Daliganirapete is identified as medicinal plants with no scientific name in WAI VAKAVITI Fijian Medicine [20]	[20]
9.	Dawa sere (dawa)	*Pometia pinnata* J.R. & G. Forst. (Sapindaceae)	[16]
10.	Denivuaka	*Sida retusa* Linn. (Malvaceae)	[19]
11.	Doi	*Alphitonia zizyphoides* (Spreng.) A. Gray (Rhamnaceae)	[16]
12.	Drala	*Erythrina variegata* L. [syn. E. *indica* Lam.] (Fabaceae)	[16]
13.	Drau ni losilosi	*Ficus scabra* G. Forst. (Moraceae)	[19]
14.	Drau ni molikaro	(unidentified) Drau ni molikaro is identified as medicinal plants with no scientific name in WAI VAKAVITI Fijian Medicine [20]	[20]
15.	Drauni baigani	*Solanum melongena* L. (Solanaceae)	[16]
16.	Drau ni danidani	*Polyscias fruticosa* (L.) Harms. (Araliaceae)	[16]
17.	Drauni kura (Kura)	*Morinda citrifolia* L. (Rubiaceae)	[16]
18.	Drauni lolo	(unidentified) Drauni lolo might be same with lolo described in Fijian Medicinal Plants [16]	[16]
19.	Drau ni quawawa	*Psidium guajava* L. (Myrtaceae)	[16]
20.	Drau ni totowiwi	*Oxalis corniculata* L. (Oxalidaceae)	[16]
21.	Drauni molautagane	*Glochidion vitiense* (Mull.Arg.) Gillespie (Phyllanthaceae)	[17]
22.	Guava	*Psidium guajava* L. (Myrtaceae)	[16]
23.	Kalabuci	*Acalypha wilkesiana* f. circinata (A. Gray) Muell. (Euphorbiaceae)	[16]
24.	Katai	*Solanum torvum* Swartz (Solanaceae)	[16]
25.	Kavika vovo	(unidentified) Kavika vovo is determined as unidentified species in Fijian Medicinal Plants [16]	[16]
26.	Lamere	(unidentified)	–
27.	Losilosi (Lose)	*Ficus barclayana* (Miq.) Summerhayes Or *Ficus pritchardii* Seem. (Moraceae)	[16]
28.	Matabulabula (Ai-rogorogo)	*Commelina diffusa* Burm. f. [syn. C. *pacifica* Vahl.] (Commelinaceae)	[16]
29.	Mokomoko	*Oberonia glandulosa* Lindl. [syn. O. *equitans* Forst. F.] (Orchidaceae)	[16]
30.	Molau [yellow]	*Glochidion bracteatum* Gillespie (Euphorbiaceae)	[16]
31.	Molau [white]	*Glochidion seemanni* Muell. Arg. (Euphorbiaceae)	[16]
32.	Molau [red fruits]	*Glochidion concolor* Muell. Arg. (Euphorbiaceae)	[16]
33.	Moli karokaro	*Citrus limon* (L.) Burm. f. (Rutaceae)	[16]
34.	Niu damu	*Cocos nucifera* L. (Arecaceae)	[16]
35.	Qatima	*Urena lobata* L. (Malvaceae)	[16]
36.	Qiqila (Sawaqa)	*Micromelum minutum* (Forst. F.) Seem. (Rutaceae)	[16]
37.	Rogo mi (Rogomi, waci ni vanua, Sewaci)	*Rorippa sarmentosa* (DC.) Macbride [syn. *Nasturtium sarmentosum* (Soland. ex Forst. f.) O.E. Schulz; *Cardamine sarmentosa* Soland. ex Forst. f.] (Brassicaceae)	[16]
38.	Taro (Dalo)	*Colocasia esculenta* (L.) Schott (Araceae)	[16]
39.	Tavotavo	*Macaranga graeffeana* Pax & K. Hoffm. (Euphorbiaceae)	[16]
40.	Tiri	*Rhizophora samoensis* (Hochr.) Salvoza [syn. R. *mangle* L., R. *mucronata* Lam.] (Phizophoraceae)	[16]
41.	Tokatoka ragigi	(unidentified)	–
42.	Tokatolu (Wavue)	*Vigna marina* (Burm.) Merr. (Fabaceae)	[16]
43.	Tonolea	(unidentified)	–
44.	Totodro	*Centella asiatica* Urban [syn. *Hydrocotyle asiatica* L.] (Apiaceae)	[16]
45.	Uci (Rauvula)	*Euodia hortensis* J.R. & G. Forst. Forma *hortensis* A.C. Sm. [syn. *Fagara euodia* Forst.] (Rutaceae)	[16]
46.	Uto	*Artocarpus altilis* (Parkinson) Fosberg (Moraceae)	[16]
47.	Vativati (Kadakada)	*Phymatosorus scolopendria* (Burm.) Pichi Sermolli [syn. *Microsorium scolopendrium* (Burm.) Copel.; *Polypodium scolopendrium* Burm.] (Polypodiaceae)	[16]
48.	Vesi (Vesi dina)	*Intsia bijuga* (Colebr.) Kuntze [syn. *Afzelia bijuga* A. Gray] (Caesalpiniaceae)	[16]
49.	Vesiwai (Vesi ni wai)	*Pongamia pinnata* (L.) (Fabaceae)	[16]
50.	Viavia	*Crinum asiaticum* L. (Amaryllidaceae)	[16]
51.	Vulokaka (Dralakaka)	*Vitex trifolia* L. var. *bicolor* (Willd.) Moldenke (Lamiaceae)	[16]
52.	Wabosucu (Ovacia)	*Mikania micrantha* H.B. & K. (Asteraceae)	[16]
53.	Wadamu (Wabula, Tolo ni wadamu)	*Merremia peltata* (L.) Merrill [syn. *Ipomoea peltata* (L.) Choisy] (Convolvulaceae)	[16]
54.	Wadenimana (Denimana)	*Dalbergia candenatensis* (Dennst.) Prain [syn. D. *monosperma* Dalz.] (Fabaceae)	[16]
55.	Walenaqio	*Psychotria archboldiana* Fosberg (Rubiaceae)	[16]
56.	Wasovivi	*Ipomoea cairica* (L.) (Convolvulaceae)	[16]
57.	Weleti (Wi, Maoli)	*Carica papaya* L. [syn. *Papaya vulgaris* Poir.] (Caricaceae)	[16]
58.	Yamenilaione	(unidentified) Yamenilaione is identified as medicinal plants with no scientific name in WAI VAKAVITI Fijian Medicine [20]	[20]

All are commonly used in Gau Island for medicinal purposes. Many species in the table have some different Fijian local names depending on the region. Scientific name of medicinal plants collected by this study were identified using medicinal plants collection books: Fijian Medicinal Plants [16], Trees of Fiji [17], Wayside Plants of the Islands [18], Plants of the Fiji Islands [19], and WAI VAKAVITI Fijian Medicine [20]

**Table 3:** Medicinal plants used for more than one treatment in Gau Island with common usage. Many medicinal plant species are used for cough and stomach ache

No	Scientific name Fijian local name		Wounds/symptoms	Common usage
1	*Ageratum conyzoides* L Botebotekoro	4	Skin boil, Cough, Stomach-ache, Stomach bloated	Anti-inflammatory, antinociceptive agents [23], cognitive enhancing effects [24], infected wounds and antitetanic [26]
2	*Bidens pilosa* L Batimadramadra	2	Cough, Asthma	Preventing diabetes [29, 30]
3	*Polyscias fruticose* (L.) Danidani	2	Stomach-ache, Sun stroke	Mmale fertility [31]
4	*Morinda citrifolia* L Drauni kura	2	A lump under armpit, Stomach-ache	Triterpenic glycoside [35]
5	*Oberonia glandulosa* Mokomoko	2	Asthma, Stomach-ache	It is reputed to be useful in alleviating pains in the chest and back, for adults. This small orchid has a flower that is imitative of a lizard, and is said to be good for pain in the lungs and back [36]
6	*Centella asiatica* Totodro	3	Cough, Asthma, Stomache-ache	Anti-inflammatory and antinociceptive agents [23], and cognitive enhancing effects [24]
7	*Rhizophora samoensis* Tiri	2	Cough, Disease of the tongue	It has reportedly been used to treat angina, boils, and fungal infections [37]
8	*Mussaenda raiateensis* Bovo (Vobo)	2	Asthma, Headache	It is positive for antimicrobial activity [38]
9	*Phymatosorus scolopendria* Vativati	2	Asthma, Stomach-ache	Strengthen mothers after childbirth [16]
10	*Vitex trifolia* L Vulokaka	3	Internal piles, Sore throat, Toothache	It exhibited moderate inhibiting activity against both gram-positive and gram-negative bacteria [39]
11	*Mikania micrantha* Wabosucu	2	Incisura, Stomache-ache	The leaves are considered to be a remedy for irritation of the skin and to sooth the sharp pain occasioned by the stings of hornets, bees, etc. [40]
12	*Dalbergia candenatensis* Wadenimana	2	Backache, Boils from inside	Antidiarrhoeal [42], and anti-inflammatory [33]
13	*Carica papaya* L Weleti	2	Incisura, Toothache	Minerals and vitamins, carotenoids, polyphenols, ABTS and CAA50 [41]

The most commonly used plant families recorded in the survey were Fabaceae (9%), Euphorbiaceae (9%), Asteraceae (7%) and Moraceae (7%), and two medicinal plants, Botebotekoro (*Ageratum conyzoides*) and Totodro (*Centella asiatica*) were used in all districts. Botebotekoro is a commonly cultivated annual herb that grows throughout the tropics [22], and was used in Qarani, Navukailagi (*Tikina Navukailagi*), Vanuaso, Lamiti (*Tikina Vanuaso*), and Vadravadra (*Tikina Sawaieke*). Totodro was used in Navukailagi (*Tikina Navukailagi*), Lekanai, Lamiti (*Tikina Vanuaso*), and Lovu, Levuka-i-Gau (*Tikina Sawaieke*). These two medicinal plant species are available throughout the island and are considered to be effective for the treatment of different ailments. According to Cambie and Ash [16], these two species are also commonly found and widely used in Fiji and many of the Pacific islands, including Tonga, Niue, Australia, and parts of Asia. Botebotekoro is used for stomach ache, skin boil, stomach bloating and cough, and Totodro is used to treat stomach ache, cough, and asthma on the island; They have scientifically proven effectiveness respectively as anti-inflammatory and antinociceptive agents [23] possessing cognitive enhancing effects [24, 25]. Botebotekoro leaf has also reportedly been used in the treatment of infected wounds and as an antitetanic in the Rewa district on the main island [26], while the flower and stem oils have been scientifically proven to be effective antibacterial agents [27]; therefore the use of Botebotekoro on the island is scientifically reasonable. Conversely, the usage of Totodro in the island has not been scientifically proven as effective in improving memory, but some regions, such as China use it in the treatment of asthma [28]. In contrast, 40 medicinal plants, including Doi (*Alphitonia zizyphoides*), Drala (*Erythrina variegata*), Viavia (*Crinum asiaticum*), and Vesiwai (*Pongamia pinnata*), were used in only one village (Additional file 1: Tables S3, S4 and S5). Cambie and Ash [16] reported that these species are valued for a small number of ailments and illnesses throughout Fiji. For example, Viavia is used to treat two ailments, wounds and infections of the breast, and Doi is reputed as a cancer treatment and there are likely several distinct local forms. Hence, the present situation of medicinal plant use in Gau may be similar to the situation on Fiji.

In Lekanai, 8 out of 58 medicinal plant species were used, and another 8 out of 58 in Lovu. Both villages have similar surrounding environments; there is no pollution around the sea coasts, no feeding damage caused by pigs and cows, and they have rich natural environments with agricultural lands separating the village settlements from the native forest. The interviewees confirmed that both villages have a bountiful natural environment. Thus, the preference for the chosen plants for medicines might depend on the availability of species. Interestingly, these two villages depend on agriculture as a source of income, whereas other villages rely mainly on fisheries and livestock (such as cattle and pigs). The livestock industry is often controversial because it is suggested to cause damage and pollution to the coasts. The villages of Lekanai and Lovu chose not to keep livestock in order to protect agricultural land as well as natural habitats, which may be one of the reasons why medicinal plants are more widely available and, therefore, more commonly used in these villages.

Among the medicinal plants surveyed, 39% were tall trees, 39% herbs, 15% small trees, and 6% herbaceous plants. The most commonly used plant part was the leaves (76%), followed by the stems (25%) and roots (4%). All parts of the plant (leaves, stems, and roots) were used only from Botebotekoro. These parts were used in mixtures to treat stomach ache in Vanuaso, whereas Botebotekoro was used in combination with other species, such as Taro, Dalo (*Colocasia esculenta*), Uci (*Euodia hortensis*), Drala (*Erythrina variegata*), and Qatima (*Urena lobata*) for the same condition in other villages.

The majority of medicinal plants are commonly found in both the wild and the village (compounds in residential areas). Two medicinal plant species have been threatened because of their small population size. Kalabuchi (*Acalypha wilkesiana*), which is found in forest and vegetation areas, was used in Lekanai, where the villagers planted this species in residential areas to conserve the population. Wadenimana (*Dalbergia candenatensis*) grow in mangrove or sea coastal areas and was used in Lamiti and Lovu. Although this plant population was commonly found in Lamiti, it was relatively rare in Lovu.

### Treatment and wounds/symptoms

The most commonly mentioned applications for medicinal plants were in the treatment of stomach ache (62%), followed by headache (25%) and wounds (25%) (Table 4).

**Table 4:** Remedies for different wounds and symptoms by medicinal plants in different villages

No	Wounds/symptoms	Number of villages	Villages
1	Asthma	1	Vione
2	Backache	2	Vione, Lamiti
3	Boils from inside	1	Lovu
4	Bone injury	1	Lovu
5	Cervix cancer	1	Nawaikama
6	Diarrheal	1	Nukuloa
7	Disease of the tongue	1	Lekanai
8	Cough	3	Navukailagi, Lamiti, Nawaikama
9	Dropsical	1	Vione
10	Fever	1	Malawai
11	Headache	4	Qarani, Nacavanadi, Lovu, Somosomo
12	Head swelling	1	Somosomo
13	Hemorrhoid	1	Levuka-i-Gau
14	Incisura	4	Navukailagi, Malawai, Lamiti, Somosomo
15	Internal piles	1	Sawaieke
16	Lump under armpit	1	Yadua
17	Runny nose and cough	1	Vanuaso
18	Sensitivity to cold	1	Lekanai
19	Skin boil	1	Navukailagi
20	Skin disease	2	Qarani, Vanuaso
21	Sore throat	1	Nukuloa
22	Stomach-ache	10	Qarani, Lekanai, Vanuaso, Nacavanadi, Malawai, Lamiti, Yadua, Lovu, Levuka-i-Gau, Sawaieke
23	Stomach bloated	1	Vadravadra
24	Sun stroke	1	Vanuaso
25	Swelling of the throat	1	Vadravadra
26	Toothache	2	Vadravadra, Nukuloa
27	Uterus cancer	1	Yadua

Thirteen plant species were used for the treatment of more than one ailment (Table 3). The medicinal herbs used to treat the highest number of ailments were Botebotekoro, used for four conditions, and Totodro and Vulokaka (*Vitex trifolia*) for three disorders. Botebotekoro is mainly used in the treatment of stomach aches, stomach bloating, coughs, and skin boils (acne). Totodro (*Centella asiatica*) is used to treat stomach ache, coughs, and asthma, and Vulokaka (*Vitex trifolia* var. *bicolor*) for sore throat, toothache, and haemorrhoids. Cambie and Ash [16] also reported that these three species are used to treat various ailments, including both less and more serious illnesses. For example, Botebotekoro is commonly employed in Fiji to treat asthma and bleeding and certain postnatal symptoms. A decoction of the leaves and stem is considered to ease menstruation, and an infusion of the plant together with the leaves and roots of vativati (*Phymatosorus scolopendria*) and the leaves of wabi (*Hoya australis*) are used to strengthen mothers after childbirth [16]. Totodro is also used to treat diarrhoea, neuralgia, and rheumatic aches and swelling, whereas some medicinal plants, such as Batimadramadra (*Bidens pilosa*), Danidani (*Polyscias fruticosa*), Matabulabula (*Commelina diffusa*) and Wadenimana (*Dalbergia candenatensis*) are used for only a few conditions. For instance, Batimadramadra is used for cough and asthma, Danidani for stomach ache and sun stroke, Matabulabula only for stomach ache, and Wadenimana for backache and internal boils. All of those medicinal plants are widely found in Asia and Africa and have been scientifically researched. A compound has been extracted from Batimadramadra for the prevention of diabetes [29, 30]. Danidani and Matabulabula potentially contribute to fertility in men [31] and infertility in women [32] respectively, and Wadenimana has been studied as an antidiarrhoeal [42], and anti-inflammatory agent [33], and if also used for other treatments.

### Unique development of medicinal plant use

There were different preparations and different combinations of plants used to treat various ailments (see Additional file 1: Tables S3, S4 and S5). In each village, often with alternative medicinal plants chosen to treat the same symptoms. For instance, in Qarani, treatment of stomach ache required four medicinal plant species: Danidani (*Polyscias fruticosa*), Taro (*Colocasia esculenta*), Matabulabula (*Commelina diffusa*), and Botebotekoro (*Ageratum conyzoides*) (see Additional file 1: Table S3). However, in Nacavanadi, Uci (*Euodia hortensis*), Vativati (*Phymatosorus scolopendria*), Mokomoko (*Oberonia glandulosa*), and Wasovivi (*Ipomoea cairica*) were employed to treat stomach ache. In addition, as shown in Additional file 1: Tables S3, S4 and S5, the process of prescribing was distinct among villages. In general, all plants collected within the village and surrounding areas were used by the inhabitants of the village. This was the case for all villages, except for Lekanai. This indicates that the status and knowledge of medicinal plants vary among villages, mainly due to geographical and social barriers between the villages. For instance, Lekanai and Vione are physically close, and the inhabitants interact. The interviewee in Lekanai is often asked for medicinal plants by Vione villagers. Lekanai and Vione have a good relationship, even though they belong to different districts, and they often have combined village meetings. Hence, in Vione, it is possible that traditional knowledge about medicinal plants and treatments for less serious diseases such as stomach ache and headache have disappeared. However, there is no exchange between Lekanai and Vanuaso which is another neighbouring village of Lekanai, which belongs to the same district but is located 1 hour away by foot.

Another possible reason for these differences among villages is social barriers. Villagers are often unaware of what medicinal plants are used in other villages, as they stated that information about important medicinal plants is understood as a traditional issue. Furthermore, the economic state of each village provides inhabitants with differing level of choice with regard to accessing modern chemical medicines. Nacavanadi village, which is called ‘the town’ on Gau, has a growing economy and villagers less frequently use traditional medicinal plants. According to the interviewees, they normally do not use medicinal plants, and chemical drugs are available for use in simple treatments as medical providers can secure transportation to the Qarani medical centre quickly. During the survey, the interviewees from Nacavanadi could only remember two traditional remedies with six medicinal plants, while the interviewees in Lekanai reported nine medicinal plants and treatments for five ailments. Thus, there are differences in the use of medicinal plants among villages depending on resource management in both social and ecological aspects.

### Social and ecological factors associated with medicinal plant use

Social and ecological factors such as geographical, environmental, cultural, and economic aspects can be barriers or facilitators in the use of medicinal plants on Gau, which could influence the inheritance of traditional knowledge and efficiency of the TRM system, as the rejection of biomedicine and continued use of healing traditions has at times been an important part of sustaining cultural identity in the face of rapid globalisation [2]. Singh [34] investigated the value of herbal medicines on Fiji and explored the reasons why traditional healers were preferred over doctors, for factors including availability, accessibility, and reliability, as well as the cost and benefit and the receipt of instruction from traditional healers; the fact that that modern medicine has no cure for certain ailments such as jaundice also played a role. Although all these social factors could explain Gau’s situation, our observation found that these explanations are closely related to each other, and there is interaction between them.

An interesting result arising from this survey is that people from Qarani, which is at the centre of the island and has the only medical centre, stated that traditional treatments using medicinal plants are more effective than modern chemical treatments for less serious illnesses. However, people from other villages that are far from the medical centre tend to believe that modern chemical treatments are better than traditional medicinal plants, and that there is no scientific evidence for treatments using medicinal plants. This may be because of different values. In the case of Qarani, people feel a sense of security by living close to the medical centre (they are able to access both the medical centre and medicinal plants at any time), and therefore they have the mental stability to make use of traditional treatments. In this case, Phillips [2] explained that the people first adopted pharmaceutical method, and then turn to medicinal plants if there is no perceptible benefit from the medication. Conversely, people living far from the medical centre, such as Vadravadra, may seek modern drugs which are not readily available to them, because people tend to desire items which are currently not attainable to them. The distance from the medical centre or nursing stations among villages may also be related to access to traditional medicine (i.e. traditional treatments tend to be more available in villages that are further away from the medical centre and the nurse stations). Phillips [2] states that people in villages far from the medical centres felt disadvantaged in their ability to manage their treatments compared to those who lived closer, whereas residents of neighboring villages of Vadravadra, such as Lovu and Yadua, had different opinions.

Another example is Yadua, Lovu, and Lekani which have similar characteristics in terms of population and perspective of land use; they have the smallest populations on the island and have rich natural environments. These communities chose to retain traditional land use methods to protect their environment and continued to use medicinal plants rather than the modern alternatives. These differences might indicate why some villages have been successful in implementing sustainable TRM systems for medicinal plants, because a small population allows them to build closer relationships and work together. In Lovu which is the only village where the chief acts as a healer, villagers prefer using medicinal plants over modern drugs because they are able to communicate with their traditional healers throughout the treatments. The constant conversation with the healers while the villagers are receiving treatment is important to gain a sense of security and mental relief that gives them the assurance to continue using the traditional methods. Thus, the distance between the village and the medical centre alone is not the only factor behind this assumption, and other issues such as the decision on land use and community size, are important when considering the people's preference in health treatments. These social factors can interact with each other and provide inconsistent results.

In addition, in all the villages, the interviewees were either the elder, the elder’s spouse, or the members of the elder’s family. Knowledge and skills concerning medicinal plants are important in these families, as they continue with the role of leading the community. Veitayaki [8] reported that knowledge of some medicinal plants is passed down through family lines and is not commonly known in the wider community, while information regarding other medicinal plants is more common and often communicated. This indicates that there may be a correlation between traditionally gifted leadership and the frequency of medicinal plant use. In addition, TRM of medicinal plants provides opportunities for each villager to communicate with members of the elder’s family. The TRM system on Gau also include the role of organising other people’s activities. These opportunities involve the organisation of volunteers to collect designated medicinal plant species from their habitats, wash and separate these plants, and prepare the necessary tools. Each village depends on individuals to perform certain duties to maintain the TRM system, which builds cooperation through volunteer community work. It has become particularly important to encourage the maintenance of the TRM system in order to improve productivity and promote healthy community relationships, as working in teams is more effective and increases their motivation to use the resources sustainably.

Statistical analysis conducted to determine the social and ecological factors affecting the TRM system showed that community solidarity (A6: social capital) has the potential to advance the state of the TRM system. Other factors such as population, economic value, and agricultural area, did not have any impact on the TRM, even though there were substantial differences among villages (Additional file 1: Table S2). The linear regression analysis showed a significant difference (*p* = 0.0326) between community solidarity (independent variables) and the current state of the TRM system (dependent variable), while the differences between other variables such as RS3: size of resource system (*p* = 0.8067) and RU4: economic value (*p* = 0.3836) were not significant (Table 5).

**Table 5:** Statistical result analyzing the relationship between social and ecological factors and the current state of the TRM system of medicinal plants

Dependent variables (the state of the TRM system) Unit: 0-No, 1-Yes				
Categories	Independent variables (social and ecological factors)	Estimate	Std. Error	*P* (>|*t*|)
	Intercept	− 0.241559	0.788411	0.77
Resource system (RS)	Size of resource system (agricultural areas) Unit: number	0.001708	0.006798	0.81
Resource units (RU)	Economic value Unit: 0-No, 1-Yes	− 0.301235	0.330581	0.38
Resource units (RU)	Number of units (average) Unit: number	0.151495	0.154448	0.35
Actor (A)	Population Unit: number	− 0.002960	0.002193	0.21
Actor (A)	Community solidarity (social capital) Unit: 0-No, 1-Yes	0.860723	0.347091	0.03 (*p* < 0.05)

This statistical analysis applied linear regression analysis presented significant difference (*p* = 0.0326) between community solidarity (independent variables) and the current state of the TRM system (dependent variable)

Based on the results, if the population influences the state of TRM, this tendency should be seen in the populous Nawaikama and Lamiti villages, but this was not the case in Nawaikama, which showed maintained TRM. In addition, there were differences in the economic value among the villages, such as in Vione and Lekanai, which have values for medicinal plants, but different states of TRM. This difference indicates that economic value has no impact; there is no correlation with the state of TRM, and the ecological elements, agricultural land area, and the number of medicinal plants also revealed no connection. However, it is difficult to measure correlation simply with agricultural area, because some villages are expanding their agricultural land while preserving the habitat of medicinal plants. There is also no correlation with the number of medicinal plants; medicinal plants are sought from neighbouring villages, through relatives or by using alternative plants.

Hence, only solidarity might be nurtured through community work of TRM, which can enhances the success of TRM and contribute to the sustainability of resource use of communities on Gau. This indicates that the TRM system and sustainable resource use are inseparable, since volunteer community work brings the community together.

## Conclusions

It is important to gain a clearer understanding of how TRM contributes to social and ecological systems. The results demonstrated that the use of medicinal plants on Gau has developed separately in each village due to various reasons, such as cultural background and geographical barriers. The current state of TRM of medicinal plants in each village varies, which is a consequence of various social and ecological factors. For example, access to the health centre and natural habitats where medicinal plants can be found are different for each village, and these factors potentially affect the frequency of use of medicinal plants, and therefore the current state of TRM. In addition, in recent years, the use of medicinal plants has decreased and modern medicine usage has increased due to interest from the younger generation, and consequently, traditional methods have declined in practice. These changes can result in disconnections between communities and the natural environment. The knowledge and skills concerning the use of traditional medicine and the TRM system on Gau contribute to the conservation of natural habitats, as well as to the formation of sustainable communities by enhancing community solidarity. Statistical analysis of the relationship between the current state of TRM and social and ecological factors indicated that community solidarity is the key to maintaining the TRM system, as the TRM and Gau people are intimately linked. They together contribute to maintaining the culture and traditions, including the knowledge and skills of the use of traditional medicines through the community work of the TRM system.

Thus, understanding the mechanisms and contribution of the TRM system with related social and ecological factors may lead to more sustainable communities. Further in-depth analysis of the relationship should be conducted with further key social and ecological factors to prevent the unsustainable use of natural resources on Gau.

## Supplementary Information

**Additional file 1: Table S1:** Second-tier variables of a social-ecological system, **Table S2:** Raw data of independent variables for statistical analysis, **Table S3:** Medicinal plants with prescription used in *Tikina Navukailagi*, **Table S4:** Medicinal plants with prescription used in *Tikina Vanuaso*, **Table S5:** Medicinal plants with prescription used in *Tikina Sawaieke*, **Appendix A:** Questionnaire form used for interview survey, **Appendix B:** Illustrations for evaluating of abundance level

## Data Availability

All data are presented in this article.
