# Supplementary material for: Traditional knowledge of medicinal plants on Gau Island, Fiji: differences between sixteen villages with unique characteristics of cultural value
Source: J Ethnobiol Ethnomed. 2021 Oct 11;17:58. doi: 10.1186/s13002-021-00481-w (PMC8507187; doi:10.1186/s13002-021-00481-w)
Supplement: Supplementary file 1 — Additional file 1: Table S1: Second-tier variables of a social-ecological system, Table S2: Raw data of independent variables for statistical analysis, Table S3: Medicinal plants with prescription used in Tikina Navukailagi, Table S4: Medicinal plants with prescription used in Tikina Vanuaso, Table S5: Medicinal plants with prescription used in Tikina Sawaieke, Appendix A: Questionnaire form used for interview survey, Appendix B: Illustrations for evaluating of abundance level [file 13002_2021_481_MOESM1_ESM.docx]

Table 1: Second-tier variables of a social-ecological system adapted from McGinnis and Ostrom (2014), and definitions of these variables.

| Social and ecological factors | | | |
| --- | --- | --- | --- |
| First-tier | | Second-tier | |
|  | | Independent variables | Definitions (units) |
| Ecological side | Resource System (RS) | Resource size | The physical size of the resource system: agricultural land areas (ha). |
|  | Resource Units (RU) | Economic value | Economic value of output (0: negative, 1: positive). |
|  |  | Number of units | Estimated population of medicinal plants (6-point scale). |
| Social side | Actor (A) | Number of actors | The number of actors in the actors’ group who are appropriators: village population (number). |
|  |  | Social capital | Norms (trust-reciprocity): community solidarity (0: negative, 1: positive). |
|  | | Dependent variables | Definitions |
|  |  | State of traditional resource management | TRM system of medicinal plants is not performed well or TRM system of medicinal plants is performed well (0: negative, 1: positive). |

Table 2: Raw data of independent variables for analysing the relationship between social and ecological factors and the current state of the TRM system by linear regression analysis. Economic value and community solidarity can take a value of 0 (negative) or 1 (positive) indicating a response of ‘No’ or ‘Yes’, respectively, as to whether or not there is economic value of medicinal plants and solidarity in a community.

| No | Tikina | Village | Population | Area (ha) | Average of plants | Economic value | Community solidarity | TRM |
| --- | --- | --- | --- | --- | --- | --- | --- | --- |
| 1. | *Tikina Navukailagi* | Navukailagi | 94 | 39.4 | 5.3 | 0 | 0 | 1 |
| 2. |  | Qarani | 133 | 23.5 | 4.3 | 0 | 1 | 1 |
| 3. |  | Vione | 99 | 58.8 | 5.5 | 1 | 1 | 0 |
| 4. | *Tikina Vanuaso* | Lekanai | 98 | 10.7 | 3.7 | 1 | 1 | 1 |
| 5. |  | Vanuaso | 150 | 15.3 | 4 | 0 | 0 | 0 |
| 6. |  | Nacavanadi | 170 | 4.7 | 4 | 0 | 0 | 0 |
| 7. |  | Malawai | 154 | 50.7 | 4 | 1 | 1 | 1 |
| 8. |  | Lamiti | 224 | 27.5 | 4.6 | 1 | 1 | 0 |
| 9. | *Tikina Sawaieke* | Yadua | 74 | 15.9 | 5.2 | 1 | 1 | 1 |
| 10. |  | Vadravadra | 140 | 6.3 | 5.7 | 1 | 0 | 0 |
| 11. |  | Lovu | 135 | 8.1 | 4.4 | 1 | 1 | 1 |
| 12. |  | Levuka-i-Gau | 128 | 13.5 | 4.6 | 1 | 1 | 0 |
| 13. |  | Nukuloa | 116 | 19.9 | 3.5 | 1 | 1 | 0 |
| 14. |  | Nawaikama | 292 | 52.5 | 6 | 0 | 0 | 0 |
| 15. |  | Somosomo | 117 | 10.5 | 5.7 | 1 | 1 | 1 |
| 16. |  | Sawaieke | 164 | 22.4 | 4.7 | 0 | 0 | 0 |

Table 3: Medicinal Plants in *Tikina Navukailagi*

| Qarani (*Tikina Navukailagi*) | | | | | | | | | | | | | | | | | | | | | | |
| --- | --- | --- | --- | --- | --- | --- | --- | --- | --- | --- | --- | --- | --- | --- | --- | --- | --- | --- | --- | --- | --- | --- |
| No. | | | Plant Name (Fijian) | | | | | Type | | | | | | Which part | | | | | Location (Abundance) | | Prescription | |
| Sickness:　Mosiniulu (kuita) (Headache) | | | | | | | | | | | | | | | | | | | | | | |
| 1 | Bovo, Vobo, Boboalewa, Bobotagane | | | | | | Tree | | | | | | | Stem | | | | | Ve(A), F(A) | | 1. Strip skin of stem (all stem). 2. Squeeze materials 1 with cold water of 2~3 tea spoon to extract the juice. Note: Do not take any of tea and cava for four days after taking this medicinal plant | |
| 2 | Walenaqio | | | | | | Tree | | | | | | | Stem | | | | | Ve(A), F(A) | |  |  |
| 3 | Doi | | | | | | Tree | | | | | | | Stem | | | | | Ve(A), F(A) | |  |  |
| Sickness: Mosinikete (Stomach-ache) | | | | | | | | | | | | | | | | | | | | | | |
| 1 | | | | Danidani | | | | | Tree | | | | | Leaf | | | | | Vi(A) | | 1. Cut five leaf from each tree. 2. Squeeze materials 1 with cold water of 3~4 tea spoon to extract the juice. Note: *There are the two types species of Danidani (plant1). | |
| 2 | | | | Taro, Dalo | | | | | Plant  (herb) | | | | | Leaf | | | | | S(C) | |  |  |
| 3 | | | | Matabulabula, Cobulabula, Drano, Ai-rogorogo, Ai-rorogi | | | | | Plant  (herb) | | | | | Leaf | | | | | Vi(A), Ve(A), F(A) | |  |  |
| 4 | | | | Botebotekoro | | | | | Tree  (herb) | | | | | Leaf | | | | | Vi(C), Ve(C) | |  |  |
| Sickness: Mosiniulu (Pini) (Early-morning headache) | | | | | | | | | | | | | | | | | | | | | | |
| 1 | | | | Yamenilaione | | | | | Tree | | | | Leaf | | | | | | Vi(A) | | | 1. Roast one leaf to make leaf soft. 2. Squeeze material 1 without any water to extract the juice. 3. Droop the juice into ear. |
| Sickness: Soso (Skin disease) | | | | | | | | | | | | | | | | | | |  | | | |
| 1 | | Viavia | | | | | | Tree  (herb) | | | | | Stem | | | | | Vi(A) | | | 1. Take off inside stem, only white part, by strip skin of stem. 2. Pound material 1 until it becomes soft and makes the juice. 3. Supply material 2 with leaf together | |
| Vione (*Tikina Navukailagi*) | | | | | | | | | | | | | | | | | | | | | | |
| Sickness: Kasi (Backache) | | | | | | | | | | | | | | | | | | | | | | |
| 1 | | | | Vesiwai, Vesi ni wai | | | | | Tree | | Stem | | | | | Ve | | | | | 1. File off skin (all skin). 2. Squeeze materials 1 with one cup of cold water to extract the juice  Note: *There are many kinds of Uto, it uses only Uto buco. *Take twice per a day, before break first and going to bed, continues for four days | |
| 2 | | | | Dawa sere, Dawa | | | | | Tree | | Stem | | | | | Ve(A) | | | | |  |  |
| 3 | | | | Uto, and many other local names | | | | | Tree | | Stem | | | | | Vi(A), Ve(A) | | | | |  |  |
| Sickness: Ceguoca (Asthma) | | | | | | | | | | | | | | | | | | | | | | |
| 1 | | | | Katai | | | | | Tree | | | Leaf | | | | | Ve(V) | | | | 1. Squeeze around ten leaf with cup of cold water to extract the juice. Note: *Take it three times per a day. | |
| Sickness: Bukete vatu (Dropsical) | | | | | | | | | | | | | | | | | | | | | | |
| 1 | | | | Moli karokaro | | | | | Tree | | Stem | | | | | | | Vi(V), Ve(V), F(V) | | | 1. Strip skin of stem of opposite side changed in color by sun heat. 2. Shave all stem. 3. Squeeze materials 2 with cold water to extract the juice. Note: *Take it three times per a day for four days. | |
| Navukailagi (*Tikina Navukailagi*) | | | | | | | | | | | | | | | | | | | | | | |
| Sickness:　Vuvu (Kid and Baby) (Cough) | | | | | | | | | | | | | | | | | | | | | | |
| 1 | | | | Totodro | Plant(herb) | | | | | | | | | | Leaf | Vi(V), Ve(V) | | | | | 1. Put each 20~30 leafs together. 2. Squeeze materials 1 without any water to extract the juice. Note: * This can be used for only baby and kids. | |
| 2 | | | | Batimadramadra | Plant(herb) | | | | | | | | | | Leaf | Vi(V), Ve(V) | | | | |  |  |
| Sickness: Bo (Skin boil) | | | | | | | | | | | | | | | | | | | | | | |
| 1 | | | | Botebotekoro | | Plant | | | | | Leaf | | | | | Vi(C) | | | | | 1. Take four leafs of Plant 1 and Squeeze. 2. Open leaf of Plant 2 and scoop gel out from inside. 3. Mix together with material 1 & 2, and spread it away on gauze. 4. Cover cutting area by gauze with material 3. Note: * Change gauze with material 3 every two or three days. | |
| 2 | | | | Daliganirapete | | Plant | | | | | Leaf | | | | | Vi(C) | | | | |  |  |
| Sickness: Mavoa (Incisura) | | | | | | | | | | | | | | | | | | | | | | |
| 1 | | | | Weleti, Wi, Maoli, Papaya (Hindi) | | Tree | | | | Stem(skin) | | | | | | | | | | Ve(V) | 1. Pound to make Plant 2 soft, and put it together with one cup of skin of stem of Plant 1. 2. Squeeze material 1 without any water by using of gauze to extract the juice. 3. Droop the juice in cuts | |
| 2 | | | | Niu damu | | Tree | | | | Stem(skin) | | | | | | | | | | Vi(V), Ve(V), F(V) |  |  |

Vi: village, Ve: vegetation areas, F: forest, S: sea coast, M: mangrove

Table 4: Medicinal Plants in *Tikina Vanuaso*

| Lekanai (*Tikina Vanuaso*) | | | | | | | | | | | | | | | |
| --- | --- | --- | --- | --- | --- | --- | --- | --- | --- | --- | --- | --- | --- | --- | --- |
| No. | Plant Name (Fijian) | | | | Type | | | | Which part | | | | Location (Abundance) | | Prescription |
| Sickness: Eshmar (Adult) | | | | | | | | | | | | | | | |
| 1 | | Tokatolu, Wavue, Drautolu | | | | Plant | | | | Leaf | | | Ve(C) | | 1. Strip skin of stem of Bovo. 2. Wrap material 1 with Tokatolu leaf. 3. Squeeze material 2 with cold water to extract the juice. Note: *Bovo has two colour flower, white and yellow. |
| 2 | | Bovo, Boboalewa, Bobotagane, Bobo | | | | Small tree | | | | Skin | | | Ve(O), F(O) | |  |
| Sickness: Eshmar (Kid) | | | | | | | | | | | | | | | |
| 1 | | Totodro | | Plant(herb) | | | | | | Leaf | | | Vi(C) | | 1. Mix all kinds of leaf. 2. Squeeze material 1 with cold water to extract the juice. Note: *Three different species of Mokomoko, only one species can be used. |
| 2 | | Vativati, Kadakada | | Plant | | | | | | Leaf | | | Vi(C), Ve(C), F(C) | |  |
| 3 | | Mokomoko | Plant(Orchid) | | | | | | | | Leaf | | F(O) | |  |
| 4 | | Batimadramadra | | Plant(herb) | | | | | | Leaf | | | Vi(C), Ve(C), F(C) | |  |
| Sickness: Stomach-ache | | | | | | | | | | | | | | | |
| 1 | | Kalabuci | | | | Plant | | | | Leaf | | | Vi(C), Ve(U), F(U) | | 1. Squeeze four leaf with half cup of warm water to extract the juice. Note: *Only red colour leaf can be used. |
| Sickness: Macake (baby:9 month ~ 1 year) (Disease of the tongue) | | | | | | | | | | | | | | | |
| 1 | | Drauni molautagane | | | | Small tree | | | | Leaf | | | Vi(C), Ve(C), F(C) | | 1. Toss six or eight leaf into mouth to extract the juice. 2. Give a teaspoon of the extracted juice to the baby. Note: *This medicinal plant effect on the condition when baby takes too much sugar, their tongue becomes white colour. |
| Sickness: Macake (kid:1~5or6 year) (Disease of the tongue) | | | | | | | | | | | | | | |  |
| 1 | | Tiri | | | | Tree | | | | Root | | | M(C) | | 1. Strip skin of four root of Tiri. 2. Pound to make material 1 soft. 3. Mix material 2 with cup of boiling water to extract the juices. Note: * This medicinal plant effect on the condition when 1~5or6 years old kids take too much sugar, their tongue becomes white colour.. |
| Vanuaso (*Tikina Vanuaso*) | | | | | | | | | | | | | | | |
| Sickness: Cevucevulala (Kid) (Skin disease) | | | | | | | | | | | | | | | |
| 1 | | Losilosi, Lose | | | | | | Tree | | Leaf | | | Vi(C) | | 1. Squeeze leaf with cold water. 2. Supply material 1 on skin. Note: * Two different species of Losilosi. |
| Sickness: Pini (Sun stroke) | | | | | | | | | | | | | | | |
| 1 | | Danidani, (gau:danidani matailalai) | | | | | | Small tree | | Leaf | | | Vi(C) | | 1. Squeeze only leaf to extract the juice. 2. Supply the juice provided by leaf on the side of nose, which you feel pain. |
| Sickness: Runny nose and cough (kid) | | | | | | | | | | | | | | | |
| 1 | | Tiri | | | | | | Tree | | Root | | | M(C) | | 1. Strip skin of root. 2. Pound to make material 1 soft. 3. Squeeze material 2 with 2~3 tea spoonful of boiling water to extract the juice. |
| Sickness: Diarrhea (Stomach-ache) | | | | | | | | | | | | | | | |
| 1 | | Botebotekoro | | | | | | Plant | | Leaf, Root & Stem | | | Vi(C) | | 1. Put all together in to cup with water. 2. Squeeze materials 1 to extract the juice. |
| Nacavanadi (*Tikina Vanuaso*) | | | | | | | | | | | | | | | |
| Sickness: Mosi ni ulu (Headache) | | | | | | | | | | | | | | | |
| 1 | | Wadamu, Tolo ni wadamu | | | | | | Plant | | Leaf | | | Ve(C), F(C) | | 1. Squeeze all leaf with a half cup of cold water to extract the juice. Note: *This headache occurs only in day time. |
| 2 | | Drauni lolo | | | | | | Tree | | Leaf | | | Ve(C), F(C) | |  |
| Sickness: Mosi ni kete (Stomach-ache) | | | | | | | | | | | | | | | |
| 1 | | Uci | | | | Plant | | | | | | Leaf | Vi(C), Ve(C) | | 1. Squeeze each four leaf with a cup of hot water to extract the juice. |
| 2 | | Vativati, Kadakada | | | | Plant | | | | | | Leaf | Vi(C), Ve(C) | |  |
| 3 | | Mokomoko | | | | Plant(Orchid) | | | | | | Leaf | Vi(C), Ve(C), F(C) | |  |
| 4 | | Wasovivi | | | | Plant(vine) | | | | | | Leaf | Vi(C), Ve(C), F(C) | |  |
| Malawai (*Tikina Vanuaso*) | | | | | | | | | | | | | | | |
| Sickness: Katakata (Kids & baby) (Fever) | | | | | | | | | | | | | | | |
| 1 | | Uci (Gau), Rauvula | | | | | | small tree | | Leaf & stem | | | Vi(C), Ve(C), F(C) | | 1. Strip skin of stem. 2. Squeeze material 1 and leaf with 2~3 tea spoonful of boiling water to extract the juice. |
| Sickness: Coka (Stomach-ache) | | | | | | | |  | |  | | |  | |  |
| 1 | | Guava | | | | | | Small tree | | Leaf & stem | | | Vi(C), Ve(C), F(C) | | 1. Strip skin of stem. 2. Squeeze material 1 and leaf with hot water to extract the juice. Note: *Take it before meals. |
| Sickness: Maboa (Incisura) | | | | | | | | | | | | | | | |
| 1 | | Wavosucu | | | | | Climber | | | Leaf | | | Vi(C), Ve(C), F(C) | | 1. Squeeze leaf without water. 2. Supply material 1 on a wound. |
| Lamiti (*Tikina Vanuaso*) | | | | | | | | | | | | | | | |
| Sickness: Vu (baby) (Cough) | | | | | | | | | | | | | | | |
| 1 | | Batimadramadrama | | | | Plant | | | | Leaf | | | Vi(U), Ve(A), F(U) | | 1. Squeeze about ten leaf without water to extract the juice. |
| 2 | | Totodro | | | | Climber | | | | Leaf | | | Vi(A), Ve(A) | |  |
| 3 | | Botebotekoro | | | | Plant | | | | Leaf | | | Vi(A), Ve(A) | |  |
| Sickness: Kasi (Backache) | | | | | | | | | | | | | | | |
| 1 | | Kavika vovo | | | | | Tree | | | Stem(skin) | | | | Vi(C), Ve(C), F(C) | 1. Strip skin of stem of Kavika bovo. 2. Wrap material 1 and stem of Wadenimana by cloth fabric. 3. Squeeze material 2 with a cup of hot water to extract the juice. Note: * Take it two or three times per a day before meals for four days. |
| 2 | | Wadenimana, Denimana | | | | | Climber | | | | Stem | | | M(C) |  |
| Sickness: Coka (Stomach-ache) | | | | | | | | | | | | | | | |
| 1 | | Drau ni quawawa | | | | | | Tree | | Leaf | | | Vi(A), Ve(A), F(A) | | 1. Squeeze each about ten leaf with a cup of hot water to extract the juice. |
| 2 | | Botebotekoro tagine | | | | | | Tree | | Leaf | | | Vi(A), Ve(A) | |  |
| Sickness: Mavoa (Incisura) | | | | | | | | | | | | | | | |
| 1 | | Wabosucu, Ovacia | | | | | Climber | | | | Leaf | | Vi(A), Ve(A) | | 1. Squeeze about each 4~5 leaf. 2. Squeeze material 1 with salt to extract the juice. 3. Supply the juice on a wound, and cover it by leaf. Note: *Change medicinal plant twice per a day. |
| 2 | | Batimadramadra | | | | | Tree(herb) | | | | Leaf | | Vi(A), Ve(A) | |  |

Vi: village, Ve: vegetation areas, F: forest, S: sea coast, M: mangrove

Table 5: Medicinal plants in *Tikina Sawaieke*

| Yadua (*Tikina Sawaieke*) | | | | | | | | | | | |
| --- | --- | --- | --- | --- | --- | --- | --- | --- | --- | --- | --- |
| No. | Plant Name | | | Type | | | Which part | | Location (Abundance) | | Prescription |
| Sickness: Mosini Kete (Stomach-ache) | | | | | | | | | | | |
| 1 | Lamere | | | Plant | | | Leaf | | S(V) | | 1. Mix all kinds of leaf (plant 1 & 2 (full of both hands (30~40 leafs))) with one cup of warm water. Note: *Take it three times per a day before meals. * It for adult and kid use |
| 2 | Denivuaka | | | Small tree | | | Leaf | | Vi(V) | |  |
| Sickness: Beka (A lump under armpit) | | | | | | | | | | | |
| 1 | Drauni kura (Gau), Kura | | | Tree | | | Leaf | | Vi(A), Ve(O) | | 1. Cut leafs into little pieces. 2. Mix Material 1 with a half cup of coconut virgin oil. 3. Bring Material 2 to a boil. 4. Supply Material 3 on a lump. Note: * The volume above described prescription is for one day (4 times). * Take it 4 times per a day for four days. |
| Sickness: Cancer (Prevention of uterus cancer) | | | | | | | | | | | |
| 1 | Molau (yellow) | | | Tree | | | Stem  (skin) | | Ve(A) | | 1. Mix all kinds of leafs and skin of stem with a cup of hot water, each full of both hands. 2. Squeeze material 1 to extract the juice by using a gauze. Note: * Qualifying age is from 18 to 40. * Take it once per a day in the morning after monthly menstruation |
| 2 | Molau (white) | | | Tree | | | Leaf | | Ve(A) | |  |
| 3 | Molau (red fruits) | | | Tree | | | Leaf | | Ve(A) | |  |
| Vadravadra (*Tikina Sawaieke*) | | | | | | | | | | | |
| Sickness: Mauvu (Stomach bloated) | | | | | | | | | | | |
| 1 | Uci (Gau), Rauvula | | | Tree | | | Leaf | Vi(V), Ve(A), F(V) | | | 1. Squeeze about ten leaf without water to extract the juice。Note:　* It for adult and kid use。* Take it three times per a day anytime for four days |
| 2 | Drala | | | Tree | | | Leaf | Vi(V), Ve(V), F(V) | | |  |
| 3 | Qatima | | | Tree | | | Leaf | V(V), Ve(V), F(V) | | |  |
| 4 | Botebotekoro | | | Plant | | | Leaf | Vi(V), Ve(V), F(V) | | |  |
| Sickness: Mosi ni bati (Toothache) | | | | | | | | | | | |
| 1 | Weleti, Wi, Maoli, Papaya (Hindi) | | | Tree | Stem(skin) | | | | Vi(V), Ve(V), F(V) | | 1. Strip skin of stem of Weleti. 2. Wrap material 1 by cloth fabric. 3. Squeeze material 2 with a cup of hot water to extract the juice. Note: *Gargle with it once per a day before meals. |
| Sickness: Kuita (Swelling of the throat) | | | | | | | | | | | |
| 1 | Bakani viti | | | Tree | | | Root | | Vi(A), Ve(A), F(A) | | 1. Grate root of plant 1 & 2. 2. Wrap material 1 by gauze. 3. Squeeze material 1 with a half cup of hot water to extract the juice. Note: *Take it three times per a day before meals at least for four days. |
| 2 | Vesi, Vesi dina | | | Tree | | | Root | | Vi(A), Ve(A), F(A) | |  |
| Lovu (*Tikina Sawaieke*) | | | | | | | | | | | |
| Sickness: Kuita (Headache) | | | | | | | | | | | |
| 1 | Qiqila, Tavolali, Sawaqa | | | Tree | Leaf | | | | Vi(A), Ve(A), F(A) | | 1. Chew four or five leaves of plant1 to extract the juice, and take these leafs off from the mouth. 2. Squeeze a full of one hand of plant2 with a half cup of water to extract the juice. Note: * It for adult and kid use. *Take it two times per a day before break first and dinner for two days. |
| 2 | Wadamu, Wabula | | | Plant | Stem(skin) | | | | Vi(V), Ve(V), F(A) | |  |
| Sickness: Sicini (Boils from inside) | | | | | | | | | | | |
| 1 | Wadenimana, Denimana | | | Climber | | | Stem(skin) | | | S(R) | 1. Wrap full of both hands of plant l by gauze. 2. Squeeze material 1 with a half cup of cold water to extract the juice. Note: * Take it two times per a day before break first and dinner. |
| Sickness: Roko (Stomach-ache) | | | | | | | | | | | |
| 1 | Vativati, Kadakada | Plant | | | | | Leaf | | Vi(A), Ve(A) | | 1. Mix all kinds of leafs, each full of both hands. 2. Squeeze material 1 with a cup of cold water to extract the juice. Note: *Take it two times per a day before breakfast and dinner for two days. |
| 2 | Totodro | Plant | | | | | Leaf | | Vi(A), Ve(A), F(A) | |  |
| 3 | Mokomoko | Plant  (Orchid) | | | | | Leaf | | Vi(C), Ve(C), F(C) | |  |
| 4 | Wasovivi | Climber | | | | | Leaf | | Vi(C), Ve(C), F(C) | |  |
| Sickness: Wai ni ramusu (Bone injury/fracture) | | | | | | | | | | | |
| 1 | Totodro | | Plant  (herb) | | | Leaf | | | Vi(V), Ve(V) | | 1. Mix all kinds of leafs and stem skin, each full of both hands. 2. Squeeze material 1 with a half cup of cold water to extract the juice. Note: * Take it before breakfast for four days. |
| 2 | Tavotavo | | Tree | | | Stem(skin) | | | Vi(C), Ve(C), F(C) | |  |
| 3 | Vesi, Vesi dina | | Tree | | | Stem(skin) | | | S(C), F(C) | |  |
| Levuka-i-Gau (*Tikina Sawaieke*) | | | | | | | | | | | |
| Sickness: Roko (Stomach-ache after Yagona) | | | | | | | | | | | |
| 1 | Wabosucu, Ovacia | | | Plant | | | Leaf | | Vi(V), Ve(V), F(V) | | 1. Mix all kinds of leafs, each about 20 ~ 30 leafs. 2. Squeeze material 1 with a cup of cold water to extract the juice. Note: * Take it two times per a day before breakfast and dinner. * It for adult use |
| 2 | Matabulabula, Ai-rogorogo, Ai-rorogi, Drano, Cobulabula | | | Plant | | | Leaf | | Vi(C) | |  |
| 3 | Totodro | | | Plant  (herb) | | | Leaf | | Vi(C), Ve(C), F(C) | |  |
| Sickness: Wai Sicini (Hemorrhoid) | | | | | | | | | | | |
| 1 | Tokatoka ragigi | | | Tree | | | Leaf | | Ve(C) | | 1. Strip skin of stem of plant2. 2. Wrap material 1 and plant1 by cloth fabric. 3. Squeeze material 2 with a cup of cold water to extract the juice. Note: * Take it twice per a day before breakfast and dinner. |
| 2 | Bobo | | | Tree | | | Stem(skin) | | Ve(A), F(A) | |  |
| Nukuloa (*Tikina Sawaieke*) | | | | | | | | | | | |
| Sickness: Mosi ni ni tilotilo (Sore throat) | | | | | | | | | | | |
| 1 | Vulokaka | | | Small tree | | | Leaf | | Vi(U) | | 1. Chew about ten leafs of Plant1 to extract the juice. Note: * Take it anytime twice per a day. * It for adult use. |
| Sickness: Mosini bati (Tooth ache) | | | | | | | | | | | |
| 1 | Vulokaka | | | Small tree | | | Stem | | Vi(V), Ve(V), F(V), S(V) | | 1. Strip skin of stem of plant1 (thumb size), and cut it into little pieces. 2. Put material1 into boiling water, and inhale water vapor from a cup. 3. Gargle by material2. Note: *Take it 2~3 times per a day before meals. |
| Sickness: Coka dra (Diarrheal) | | | | | | | | | | | |
| 1 | Drau ni molikaro | | | Small tree | | | Leaf | | Vi(U), Ve(C) | | 1. Roll about 5 ~ 6 leafs. 2. Chew material 1 to extract the juice in the mouth. Note: *Take it anytime 3 ~ 4 times per a day. * It for adult and kid use. * Use only young (soft) leaves |
| 2 | Drau ni losilosi | | | Small tree | | | Leaf | | Vi(U), Ve(C) | |  |
| Nawaikama (*Tikina Sawaieke*) | | | | | | | | | | | |
| Sickness: Cenisa (Cervix cancer) | | | | | | | | | | | |
| 1 | Rogo mi, Rogomi, Waci ni vanua, Sewaci | | | Plant | | | Leaf | | Vi(V), Ve(V) | | 1. Squeeze all kinds of leafs together without water. 2. Supply material 1 on a place. 3. Wash it off by warm water properly next morning. Note: *Supply it for four days. |
| 2 | Drauni totowiwi | | | Plant | | | Leaf | | Vi(V), Ve(V) | |  |
| Sickness: Vuvu (Cough) | | | | | | | | | | | |
| 1 | Tonolea | | | Small tree | | | Leaf | | Vi(V) | | 1. Squeeze about 20 ~ 30 leafs without water to extract the juice. Note: * One tea spoon for kid. * 2 ~ 3 tea spoon for adult. * Take it 2 times per a day before breakfast and dinner. |
| Somosomo (*Tikina Sawaieke*) | | | | | | | | | | | |
| Sickness: Mosi ni ulu (Headache) | | | | | | | | | | | |
| 1 | Drau ni danidani | | | Plant | | | Leaf | | Vi(V), Ve(V) | | 1. Squeeze about 20 ~ 30 leafs without water to extract the juice. 2. Drop material 1 into ear and nose. Note: * Take it twice per a day. |
| Sickness: Mavoa (Incisura) | | | | | | | | | | | |
| 1 | Wabosucu, Ovacia | | | Plant | | | Leaf  stem | | Vi(V), Ve(V) | | 1. Squeeze about 20 ~ 30 leafs and stem together without water to extract the juice . 2. Supply the juice on a wound. Note: * Hemostatic effect. |
| Sickness: Kuita (Head swelling) | | | | | | | | | | | |
| 1 | Qiqila, Tavolali, Sawaqa | | | Small tree | | | Leaf | | Vi(A), Ve(A), F(A) | | 1. Stack about 6 ~ 8 leafs in alternating layers, and roll it. 2. Chew material 1. 3. Swallow the juice. 4. Take out leafs from mouth, and supply it on head swelling. Note: *Take it anytime 2~3 times per a day. |
| Sawaieke (*Tikina Sawaieke*) | | | | | | | | | | | |
| Sickness:　Sicini (Internal piles) | | | | | | | | | | | |
| 1 | Drauni baigani (Gau), Egg plant, Aubergine, Baigani (Hindi) | | | Plant | | | Leaf | | Vi(A), Ve(A) | | 1. Squeeze 6~8 of leafs of Plant 1 with cup of cold water to extract the juice. 2. Chew a few leafs of Plant 2 to extract the juice, and take these leafs off from the mouth, then put these leafs on a boiling point. Note: * Take the each juice separately. |
| 2 | Vulokaka, Dralakaka, Mulokaka | | | Small tree | | | Leaf | | Vi(A) | |  |
| Sickness:　Roko (Stomach-ache) | | | | | | | | | | | |
| 1 | Drauni kura (Gau), Kura | | | Tree | | | Leaf | | Vi(C), Ve(C), F(C) | | 1. Squeeze 6~8 leafs with half cup of cold water to extract the juice. Note: *Take it once a day before meal. |

Vi: village, Ve: vegetation areas, F: forest, S: sea coast, M: mangrove

Appendix A.

Questionnaire form used for interview survey


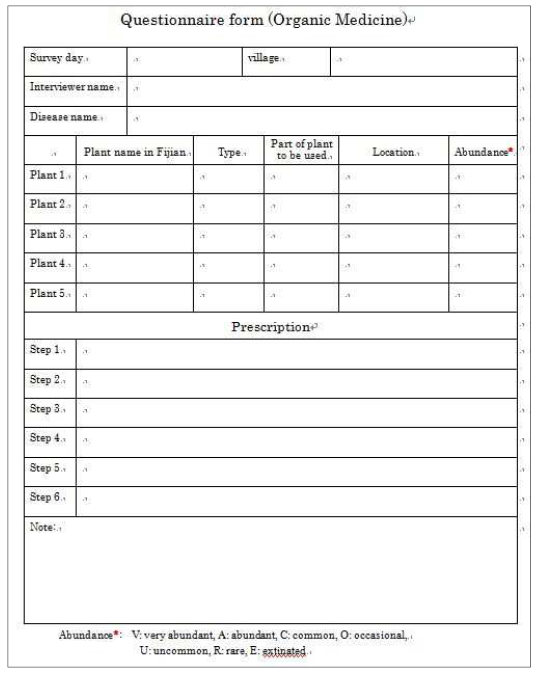


**Appendix B. Illustrations for evaluating**

Abundance level


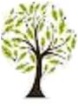

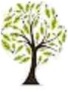

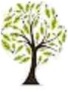

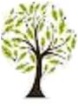

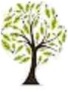

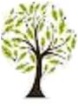

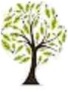

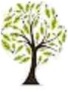

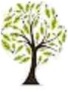

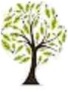

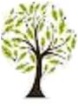

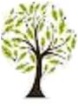

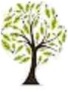

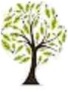

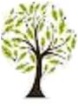

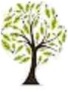

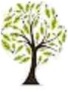

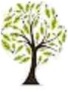

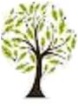

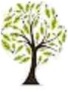

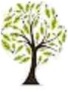

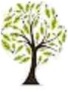

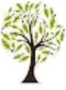

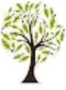

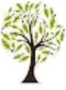

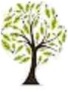

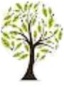

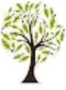

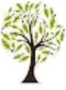

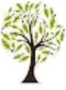

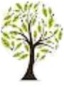

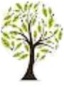

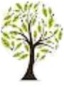

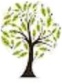

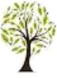

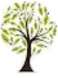


V: very abundant

A: abundant

C: common

O: occasional

U: uncommon

R: rare
